# The architecture of clonal expansions in morphologically normal tissue from cancerous and non-cancerous prostates

**DOI:** 10.1186/s12943-022-01644-3

**Published:** 2022-09-22

**Authors:** Claudia Buhigas, Anne Y. Warren, Wing-Kit Leung, Hayley C. Whitaker, Hayley J. Luxton, Steve Hawkins, Jonathan Kay, Adam Butler, Yaobo Xu, Dan J. Woodcock, Sue Merson, Fiona M. Frame, Atef Sahli, Federico Abascal, CRUK-ICGC Prostate Cancer Group, Abraham Gihawi, Adam Lambert, Alan Thompson, Andrew Futreal, Andrew Menzies, Anne Baddage, Anthony Ng, Atef Sahil, Barbara Kremeyer, Bissan Al-Lazikani, Charlie Massie, Christopher Greenman, Christopher Ogden, Clare Verrill, Cyril Fisher, Dan Berney, Dan Burns, Daniel Leongamornlert, David Jones, David Nicol, David Wedge, Declan Cahill, Douglas Easton, Edward Rowe, Ekaterina Riabchenko, Elizabeth Bancroft, Erik Mayer, Ezequiel Anokian, Freddie Hamdy, Gahee Park, Gill Pelvender, Gregory Leeman, Gunes Gundem, Hongwei Zhang, Ian G. Mills, Jingjing Zhang, Jon Teague, Jorge Zamora, Katalin Karaszi, Kieran Raine, Lucy Matthews, Lucy Stebbings, Ludmil B. Alexandrov, Luke Marsden, Mahbubl Ahmed, Matti Nykter, Mohammed Ghori, Naomi Livni, Nening Dennis, Nicholas Van As, Niedzica Camacho, Nimish Shah, Pardeep Kumar, Peter Van Loo, Radoslaw Lach, Sandra Edwards, Sara Pita, Sarah J. Field, Sarah Thomas, Simon Tavaré, Stefania Scalabrino, Steven Hazell, Stuart McLaren, Tapio Visakorpi, Thomas J. Mitchell, Tim Dudderidge, Tokhir Dadaev, Ultan McDermott, Valeria Bo, Valeriia Haberland, Vincent Gnanapragasam, Vincent Khoo, William Howat, Yong Jie-Lu, Yongwei Yu, Zsofia Kote-Jarai, Iñigo Martincorena, G. Steven Bova, Christopher S. Foster, Peter Campbell, Norman J. Maitland, David E. Neal, Charlie E. Massie, Andy G. Lynch, Rosalind A. Eeles, Colin S. Cooper, David C. Wedge, Daniel S. Brewer

**Affiliations:** 1 Norwich Medical School, University of East Anglia, Norwich, Norfolk, NR4 7TJ UK; 2 Department of Histopathology, Cambridge University Hospitals NHS Foundation Trust, Cambridge, CB2 0QQ UK; 3 Cancer Research UK Cambridge Institute, Cambridge, CB2 0RE UK; 4 Molecular Diagnostics and Therapeutics Group, Division of Surgery and Interventional Sciences University College London, London, W1W 7TS UK; 5 Cancer, Ageing and Somatic Mutation, Wellcome Trust Sanger Institute, Hinxton, CB10 1RQ UK; 6 Oxford Big Data Institute, University of Oxford, Old Road Campus, Oxford, OX3 7LF UK; 7 The Institute of Cancer Research, London, SW7 3RP UK; 8 Cancer Research Unit, Department of Biology, University of York, Heslington, YO10 5DD North Yorkshire UK; 9 Faculty of Medicine and Health Technology, Tampere University and Tays Cancer Center, 33014 Tampere, FI Finland; 10 HCA Laboratories, London, WC1E 6JA UK; 11 Department of Oncology, University of Cambridge, Cambridge, CB2 0XZ UK; 12 School of Medicine/School of Mathematics and Statistics, University of St Andrews, St Andrews, KY16 9AJ UK; 13 Royal Marsden NHS Foundation Trust, London and Sutton, SM2 5PT UK; 14 Manchester Cancer Research Centre, University of Manchester, Manchester, M20 4GJ UK; 15 Earlham Institute, Norwich, NR4 7UZ UK

**Keywords:** Prostate cancer, Clonal expansions, Genomics, Normal tissue, Benign prostatic hyperplasia, Field effect, Mutational signatures

## Abstract

**Background:**

Up to 80% of cases of prostate cancer present with multifocal independent tumour lesions leading to the concept of a field effect present in the normal prostate predisposing to cancer development. In the present study we applied Whole Genome DNA Sequencing (WGS) to a group of morphologically normal tissue (*n* = 51), including benign prostatic hyperplasia (BPH) and non-BPH samples, from men with and men without prostate cancer. We assess whether the observed genetic changes in morphologically normal tissue are linked to the development of cancer in the prostate.

**Results:**

Single nucleotide variants (*P* = 7.0 × 10^–03^, Wilcoxon rank sum test) and small insertions and deletions (indels, *P* = 8.7 × 10^–06^) were significantly higher in morphologically normal samples, including BPH, from men with prostate cancer compared to those without. The presence of subclonal expansions under selective pressure, supported by a high level of mutations, were significantly associated with samples from men with prostate cancer (*P* = 0.035, Fisher exact test). The clonal cell fraction of normal clones was always higher than the proportion of the prostate estimated as epithelial (*P* = 5.94 × 10^–05^, paired Wilcoxon signed rank test) which, along with analysis of primary fibroblasts prepared from BPH specimens, suggests a stromal origin. Constructed phylogenies revealed lineages associated with benign tissue that were completely distinct from adjacent tumour clones, but a common lineage between BPH and non-BPH morphologically normal tissues was often observed. Compared to tumours, normal samples have significantly less single nucleotide variants (*P* = 3.72 × 10^–09^, paired Wilcoxon signed rank test), have very few rearrangements and a complete lack of copy number alterations.

**Conclusions:**

Cells within regions of morphologically normal tissue (both BPH and non-BPH) can expand under selective pressure by mechanisms that are distinct from those occurring in adjacent cancer, but that are allied to the presence of cancer. Expansions, which are probably stromal in origin, are characterised by lack of recurrent driver mutations, by almost complete absence of structural variants/copy number alterations, and mutational processes similar to malignant tissue. Our findings have implications for treatment (focal therapy) and early detection approaches.

**Supplementary Information:**

The online version contains supplementary material available at 10.1186/s12943-022-01644-3.

## Background

Prostate cancer is a multifocal, highly heterogeneous disease [1, 2] that is the most common cancer diagnosed in men in the world, with an estimated 50% of men over 60 having cancer present in the prostate [3]. The phenomenon of field cancerization was first described by Slaughter et al. [4] after observing the presence of multiple independent tumours in 11% of patients with oral squamous cell carcinomas. It was proposed that the areas surrounding these lesions were acting as a “field”, a preconditioned epithelium that could lead to cancer development. This theory suggests that tissue with a histomorphologically normal appearance can harbour a significant burden of mutations, early clonal expansions, distinct expression profiles and methylation changes that could potentially lead to tumour development. Numerous reports of somatic mutations and clonal expansions in aging individuals are in agreement with this theory [5–8]: there is clear evidence that somatic mutations are present in morphologically normal skin [5, 9], brain [10], liver [11], oesophagus [6, 12], and colorectum [13] – in some cases affecting cancer-associated driver genes. Comparable findings have been reported in blood, where the detection of clonal expansions in healthy patients over 65 has been associated with a significant increase in the risk of leukemia [14–17]. Somatic mutations and clonal expansions were found to be frequently present in RNA sequencing data collected from morphologically normal tissue from patients with a wide range of cancers [18]. It was found that tissues, such as skin, lung and oesophagus, that had a direct exposure to environmental carcinogenic factors (UV radiation, smoking and nutritional habits), or had a very high proliferation rate exhibited the highest mutation burden [18]. There is also some evidence that in certain situations mutant clones in normal epithelium can play an anti-tumorigenic role [19].

In prostate cancer around 70–80% of men are found to have multifocal lesions at the time of diagnosis [20], with the separate cancers having distinct genetic trajectories [21]. Many studies support the presence of field cancerization in the prostate. We previously reported [22] that clonal expansions were present in the morphologically normal tissues of three prostates from men with prostate cancer even in tissues distant from the tumour. Similarly, a higher mutation rate was observed in mitochondrial DNA from morphologically normal adjacent tissue in men with cancer in comparison to healthy controls [23]. In an in-depth examination of one prostate, somatic mutations were estimated to accumulate steadily at 16 mutations/year [24]. Different patterns in gene expression were observed in morphologically normal tissue adjacent to cancer compared to normal tissues from men without cancer [25, 26]. A similar scenario is observed when analysing methylation profiles from tumour adjacent normal tissue and normal tissue from non-cancer patients, highlighting the potential importance of methylation in prostate cancer development [27, 28].

In this study, whole genome sequencing was performed on multiple samples from morphologically normal tissues from 37 men with and without multifocal prostate cancer, to gain insights into the nature of the field effect in the prostate.

## Methods

### Sample selection and ethics

Samples were collected at prostatectomy (from men with prostate cancer) and at cystoprostatectomy (from men without prostate cancer) from the Addenbrooke’s Hospital, Cambridge, UK. Samples from men without prostate cancer were collected at autopsy at the Tissue and Research Pathology/Pitt Biospecimen Core at the University of Pittsburgh. Samples of cell cultured fibroblasts derived from stroma were collected from York Teaching Hospital NHS Foundation Trust and Castle Hill Hospital in Hull. Clinical details for the patients are presented in Additional file 1. Ethical approval was obtained from the NHS East of England-Cambridge REC [03/018] and from the NHS Hull and East Yorkshire (REC ref/07/H1304/121) for the morphologically normal samples (including BPH) and cultures, respectively. Samples were collected subject to ICGC standards of ethical consent (https://icgc.org/). Blood samples were used as normal controls apart from the fibroblast samples where cell cultured lymphocytes were used.

The prostates were processed as previously described [29]. In brief, 5 mm slices were selected for each prostate and 4–6 mm cores were taken from them and frozen. Transverse 5 μm sections were taken from the frozen cores and H&E stained and immediately adjacent 6 × 50 μm sections were used for DNA preparation. At least two histopathologists confirmed the presence or absence of cancer and percentage estimates in central pathology review of the 5 μm H&E stained tissue slices. Prostates were deemed multifocal if, in an estimated 3D reconstruction from prostatectomy slices, two nodules are clearly separated in all planes (> 2 mm apart). The distance (in mm) between all the morphologically normal samples and their respective tumours, where present, was measured.

### DNA sequencing

DNA was extracted from 121 samples from 37 participants: 37 matched blood controls, 39 morphologically normal samples from men with prostate cancer (BPH and non-BPH), 38 samples from tumour and 7 samples from men without prostate cancer (5 from autopsy and 2 from cystoprostatectomy; Table 1; Additional file 1). Additionally, DNA was extracted from an extra five samples from the passage 1 stroma cultured from morphologically normal regions with BPH, along with matched cell cultured lymphocyte controls. The cells used were true primary cultures, where the expression phenotype matched that of tissue stroma and preserved the complexity of tissue stromal phenotypes [30, 31].

**Table 1:** Summary of samples collected from morphologically normal, BPH and tumour tissues from patients with and without prostate cancer. Patients 0006, 0007 and 0008 have multiple samples from non-BPH normal and tumour tissue and patients 0065, 0073 and 0077 have a sample from non-BPH and BPH normal tissue (Supplementary Table 1). Five samples were sequenced from stroma cultured from morphologically normal regions with BPH from five cancerous prostates in a separate cohort of men

	**SAMPLES**		
**PATIENTS**	**Morphologically Normal tissue**		**Tumour tissue**
**Cancer (30)**	**Non-BPH**	**30 (Prostatectomy)**	**38 (Prostatectomy)**
	**BPH**	**9 (Prostatectomy)**	
**Cancer (5)**	**Fibroblasts**	**5 (Cell culture)**	
**Non-cancer (7)**	**Non-BPH**	**6 (1 Cystoprostatectomy and 5 Autopsy)**	
	**BPH**	**1 (1 Cystoprostatectomy)**	

DNA from whole blood samples and frozen tissue was extracted and quantified using a ds-DNA assay (UK-Quant-iT PicoGreen® dsDNA Assay Kit for DNA) following manufacturer’s instructions with a Fluorescence Microplate Reader (Biotek SynergyHT, Biotek). Acceptable DNA had a concentration of at least 50 ng/μl in TE (10 mM Tris/1 mM EDTA), with an OD 260/280 between 1.8–2.0.

Paired-end whole genome sequencing of the samples was performed at Illumina, Inc. (Illumina Sequencing Facility, San Diego, CA USA) as described previously [22]. Sequencing data from each lane was aligned to the GRCh37 reference human genome [32] using the Burrows-Wheeler Aligner’s Smith-Waterman Alignment (BWA-SW) [33] v0.5.9-r16 + rugo using parameters -1 32 -t 6. Lanes that pass quality control are merged into a single well-annotated sample BAM file with PCR duplicate reads removed. These data have been submitted to the European Genome-Phenome Archive (EGAD00001000689 and EGAD00001004125).

### Variant calling

Single nucleotide variants (SNVs), insertions and deletions were detected using the Cancer Genome Project Wellcome Trust Sanger Institute pipeline. An updated version of this pipeline is available as a Docker image (Alignment: https://dockstore.org/containers/quay.io/wtsicgp/dockstore-cgpmap; Variant-calling: https://dockstore.org/containers/quay.io/wtsicgp/dockstore-cgpwgs)﻿.

**SNVs:** somatic single nucleotide variants (SNVs) were called using CaVEMan, https://github.com/cancerit/CaVEMan). CaVEMan (Cancer Variants through expectation Maximization) is an algorithm developed at the Wellcome Trust Sanger institute to find somatic substitutions in NGS sequencing data [34]. It is a Bayesian probabilistic classifier that uses an expectation maximization (EM) algorithm. This algorithm calculates a probability score for likely phenotypes at each genomic position, given prior information regarding reference alleles, CNAs or ploidy, the fraction of aberrant tumour cells present in each cancer sample and sequencing quality scores. A high level of specificity and sensitivity was achieved by applying project specific post-processing filters [35]. These filters were designed according to previous results from visual inspection of hundreds of variants. In comparisons with other mutation callers it has been found to be amongst the top performers in terms of sensitivity and specificity [36]. Visual inspection was performed for all variants in five patients and in all SNVs affecting recurrently mutated genes, as previously described [22].

**Indels**: Insertions and deletions were called using a lightly modified version of pindel [37]

(http://cancerit.github.io/cgpPindel/).

**Structural rearrangements** were called using Brass (Breakpoints via assembly, https://github.com/cancerit/BRASS), an in-house bespoke algorithm developed at the Wellcome Trust Sanger institute to find genomic rearrangements in paired-end NGS sequencing data. In brief, the first step is to combine discordant read pairs into potential regions where a breakpoint might occur. Next, reads around each potential region, including half-unmapped reads, are gathered and a local de novo assembly using Velvet is performed [38]. By analysing the De Bruijn graph pattern, the breakpoint can be identified down to base pair resolution.

**Copy number:** clonal and sub-clonal somatic CNAs was detected with the Battenberg algorithm (https://github.com/Wedge-Oxford/battenberg) [39]. An estimation of ploidy and tumour content is estimated as previously described [39].

### Statistical analyses

All statistical analyses were implemented in R, version 3.6.1. In comparisons where multiple samples from a patient were present in a group the median value was taken.

### Mutational signatures detection

The recently published new mutational catalog [40] was used for the decomposition of mutational processes in each sample using SigProfiler (https://github.com/AlexandrovLab/SigProfilerSingleSample) as previously described [41]. Alexandrov et al. [40] confirmed all the previously reported COSMIC signatures (except for Signature 25) and added 20 more signatures. All mutational signatures from the catalogue were included in the analysis, except signature 25.

Only signatures with exposures higher than the recommended 0.06 cutoff are reported [42]. Samples with less than 100 SNVs were excluded from this analysis (0001_N, 0008_N3 and 0007_T4).

### Analysis of subclonal architecture

The subclonal architecture of normal and tumour samples from individual prostates was reconstructed using a Bayesian Dirichlet process adapted to cluster SNVs in *n* dimensions [43] as previously described [22, 43, 44] (DPClust). In those cases where there was only one sample (normal samples without a matched tumour i.e. non-cancer patients and BPH-fibroblasts) the subclonal architecture was reconstructed using a standard Dirichlet model. The fraction of cells carrying a particular mutation (clonal cell fraction) was estimated from the mutant allele fraction, copy number alterations (CNAs) and purity. In normal and BPH samples the purity is assumed to be 100%. Only those clones supported by at least 1% of total SNVs for each patient were retained. For cases 6–8, mutations that were previously validated by deep sequencing [22] were kept for the phylogeny reconstruction. In all cases the allele frequencies of the subclone were significantly different to the estimated background rate (*P* < 0.05).

### Neutral evolution tests

Neutrality analyses were performed using the R package Neutralitytestr [45]. This package uses SNV allele frequencies and fits a neutral model of evolution. In brief, the model predicts that subclonal mutations (with allele frequency < 0.25) follow a 1/f power law distribution. For these analyses, only those mutations with VAF > 0.1 were considered, the package default. Subclonal clusters were removed from further analysis when a threshold for neutrality was met (*P* > 0.05; area under the curve, Kolmogorov distance, Euclidean distance).

### Functional impact

The tool wANNOVAR^199^ was applied to assess the functional impact of our set of nucleotide variants. It analyses the position (chromosome, location, reference and alternate nucleotides) of each mutation. The COSMIC and The Human Protein Atlas database (https://www.proteinatlas.org/) were used to report cancer associated genes.

## Results

### Mutation profiles of normal tissue

We performed Whole Genome DNA Sequencing (WGS) on 39 samples of morphologically normal tissue (median depth 53.4X) and 38 samples of cancer (median depth 58.4X) taken from the prostates of 30 cancer patients (Table 1; Additional file 1). 24/30 (80%) of the patients had multifocal tumours, suggesting presence of a field effect, and nine of the morphologically normal samples were classified as coming from a region of benign prostatic hyperplasia (BPH). Multiple morphologically normal samples from the same patient were taken in six cases (Patients 0065, 0073, 0077, 0006, 0007 and 0008) (Supplementary Table 1 in Additional file 2; Additional file 1). Matched tumours were included for all patients except patient 0240. In addition, normal prostate tissue samples were sequenced from seven non-cancer patients: two collected after a cystoprostatectomy and five from samples collected at autopsy (median depth 54.6X). Matched blood controls were included for all patients. An extra five samples were sequenced from stroma cultured from morphologically normal regions with BPH from five cancerous prostates in a separate cohort of men (median depth 55.4X; matched cell cultured lymphocytes were used as controls). A total of 131 samples were analysed by WGS, of which 43 are blood controls.

**Fig. 1:**
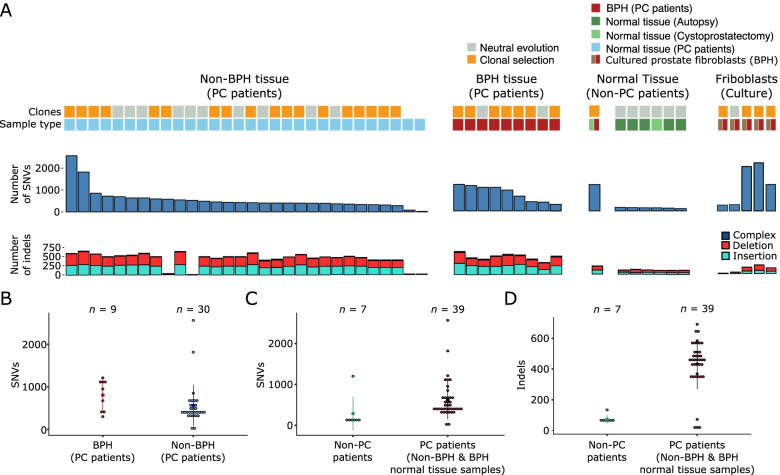
Mutations in morphologically normal tissue: **A** From top to bottom: whether clonal expansions under positive selection were detected; sample type (morphologically normal tissue in prostate cancer patients, BPH tissue in prostate cancer patients, tissue from non-prostate cancer patients, BPH fibroblast cell culture); number of single nucleotide variants (SNVs) detected per sample; number of indels (insertions, deletions and complex insertions/deletions) per sample. Each column represents a sample and they are ordered according to sample type and decreasing number of SNVs. Eight rearrangements (not represented in figure) were detected across all patients (sample 0063_N (*n* = 1), 0127 (*n* = 3), 0073_N (*n* = 1), 0074_N (*n* = 1), 0006_N1 (*n* = 1) and sample 0006_N3 (*n* = 1)). A *BRCA2* SNP (chr13:32,945,095) was detected in the blood of donor 0063. No copy number alterations were detected. **B** Plot showing the distribution of the number of SNVs found in BPH samples and non-BPH normal samples in prostate cancer patients; **C** the number of SNVs between normal samples from people with or without prostate cancer; **D** the number of indels between normal samples from people with or without prostate cancer

In morphologically normal samples, no copy number alterations and a low number of structural rearrangements (*n* = 7) were detected. In total, 26,135 Single Nucleotide Variants (SNVs) (median of 421 per sample), and 17,370 indels (median of 445) were identified in morphologically normal samples (Fig. 1). The number of mutations shared between samples from the same donor ranged from 0 to 622 SNVs (Supplementary Table 2). Cultured prostate fibroblasts also harboured a high number of SNVs (6,597 total: median of 1116), suggesting the possibility of a stromal origin for the mutations observed in normal tissue. The number of SNVs and indels were significantly higher in morphologically normal samples from men with prostate cancer compared to those without (SNVs, median 436 for cancer vs 141 non-cancer, *P* = 7.0 × 10^–03^, Wilcoxon rank sum test; and Indels, median for cancer 455 vs 62 non-cancer, *P* = 8.7 × 10^–06^, Wilcoxon rank sum test). Cystoprostatectomy sample 0239, which is classed as BPH, had an exceptionally high number of mutations (1202) in comparison to the other non-cancer patients. There is some evidence that a higher number of SNVs is present in BPH samples compared to non-BPH morphologically normal tissue (median 952 for BPH compared to 424, *P* = 0.018, Wilcoxon rank sum test).

There was no evidence of an association between the number of SNVs and the distance between morphologically normal and tumour samples (*ρ* = -0.00015, *P* > 0.99, Spearman’s correlation) or between the number of SNVs/indels and multifocality (*P* = 0.38, and *P* = 0.73, Wilcoxon rank sum test, respectively). Similarly, although age is a known contributor to prostate cancer development, no association was found between age and the number of mutations in morphologically normal samples (*ρ* = 0.26*, P* = 0.082 Spearman’s correlation; Supplementary Fig. 1 in Additional file 2). However, the age distribution is not representative of the general population. The number of SNVs were still significantly associated with prostate cancer status when age was included as a covariate (*P* = 0.018; coefficient = 362; linear model).

### Subclonal architecture

The subclonal architecture of normal and tumour samples from each individual prostate was reconstructed using the DPclust method [43] (Additional file 3; Additional file 4, Supplementary Fig. 2). Clones where there was a suggestion of neutral evolution were removed (see Methods). Subclonal architecture was supported by shared alterations including SNVs, indels and structural rearrangements.

The number of samples with subclonal expansions under selective pressure were significantly higher in morphologically normal tissue taken from cancer patients (23/37) compared to that taken from non-cancer patients (1/7 samples; *P* = 0.035, Fisher exact test; Fig. 1, Additional files 3 & 4). Clonal expansions under selective pressure were also detected in four of five fibroblasts samples (cases 0247, 0250, 0251 and 0252), where single nucleotide variants were present at clonal cell fractions (CCF) of 24%, 40%, 100% and 77% of cells, respectively (Supplementary Fig. 2, Additional file 4).

**Fig. 2:**
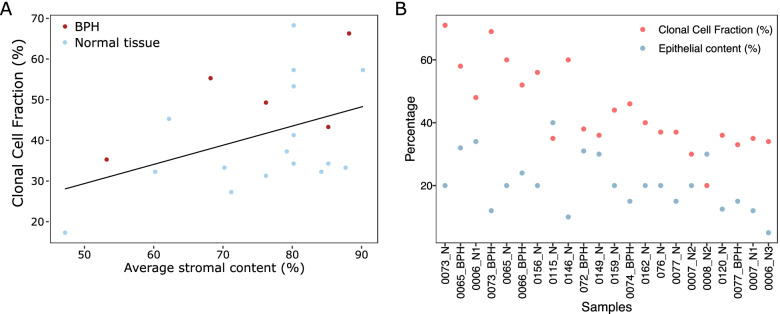
Relationship between clonal cell fraction (CCF) of clones in morphologically normal sample and estimated cellular composition. **A** Scatter plot of average stromal content estimated by histopathological review and the CCF for each morphologically normal sample from men with prostate cancer. Line is the best fit linear line. Colour is whether the sample is BPH or not. **B** Comparison between the CCF and the percentage epithelial content for each morphologically normal sample from men with prostate cancer

No significant differences were found between the CCFs of non-BPH morphologically normal (median of 37) vs BPH tissue (median of 49) samples, BPH cultured fibroblasts (median of 56.5) vs BPH tissue samples, and BPH cultured fibroblasts vs non-BPH morphologically normal samples (*P* > 0.36, Wilcoxon rank sum test, Supplementary Fig. 4). The CCF of clonal expansions of both BPH and non-BPH morphologically normal tissue was weakly associated with the stromal content (%) of each sample (*r* = 0.30, *P* = 0.16, Spearman’s correlation, Fig. 2A). More importantly, the CCF is always higher than the proportion of the prostate estimated as epithelial (Fig. 2B; median CCF = 39, median epithelial = 20; *P* = 5.94 × 10^–05^, paired Wilcoxon signed rank test, Additional file 5), which suggests that the cells containing the clonal expansions are likely to be of stromal origin.

**Fig. 3:**
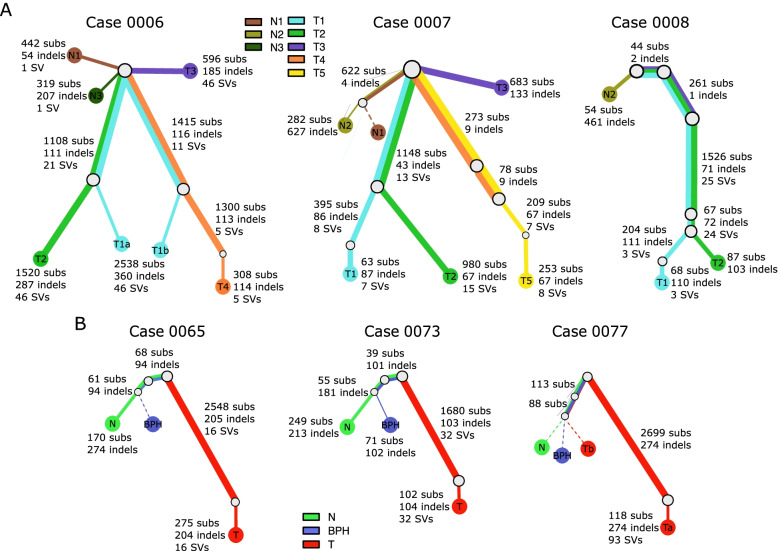
Phylogenies of patients with multiple samples. Phylogenies revealing the relationships between clones for each case. **A** patients where we have collected multiple tumours and normal. **B** patients where there was data from a tumour, non-BPH normal tissue, and BPH normal tissue. Each coloured line represents a clone/subclone detected in a particular sample. When two or more coloured lines are together, they represent a clone that is found in all the samples represented. The length of the line is proportional to the weighted number of single nucleotide variants present in each clone; the thickness represents the clonal cell fraction associated with that clone (more detail in Additional file 3). For example, case 0077 contains a shared subclone with 8% N, 33% BPH and 2% T (Tb) supported by 113 SNVs and 4 indels. Dotted lines are associated with samples that have no evidence of a unique sample specific clone. The very low fraction tumour subclone (< 4%) shared with normal and BPH tissue in case 0077 and between normal and tumour in case 0072 suggests cancer targeted tissue contained some of the N/BPH cells. Additional phylogenies can be found in Supplementary Fig. 3

To illustrate the relationship among different clones, phylogenetic trees were constructed using the sum and crossing rule [46] for 17 patients where at least one clonal expansion was detected in normal tissue (Fig. 3, Supplementary Fig. 3). In the three patients that we have examined in previous work [22], data from multiple additional morphologically normal samples was available enabling more detailed mapping (Fig. 3A). We observe that mutation clusters in normal tissue are all subclonal (Additional file 3), with a shared N1/N2 subclone in case 0007, two subclones (N1 and N3) in case 0006, and one clone in N2 in case 0008. These results show that multiple clonal expansions of morphologically normal cells are present in the prostate of some men with prostate cancer. There is no shared trunk between tumour clones and normal clones, indicating that they arise independently.

BPH and non-BPH morphologically normal tissue taken from the same prostate shared a subclone in all three cases examined (0065, 0073 and 0077, Fig. 3B). Generally, mutations present in morphologically normal tissue (BPH or non-BPH) and cancer were distinct but in case 0077 a subclone was observed with 2% contribution in the tumour sample, 8% in the morphologically normal sample and 33% in the BPH sample, consistent with a model in which the tumour sample contains a small proportion of the non-BPH/BPH subclone.

In the remaining 11 patients, where morphologically normal (either BPH or non-BPH) and tumour samples were taken, two patterns were present. The first pattern (Cases 0066, 0074, 0115, 0149 Supplementary Fig. 3) was characterised by separate cancer and non-BPH morphologically normal lineage. In the second pattern (Cases 0072, 0076, 0120, 0146, 0156, 0159, 0162) there was evidence of a subclone found in the normal cells also being present in the cancer sample at a low CCF (< 13%, median of 3, IQR of 2; Additional file 3). The minimum distance between cancer and normal samples for the prostates with independent lineages (median of 19 mm; IQR = 9) was on average larger than prostates where the cancer samples had a normal clone present (median 7.1 mm; IQR = 5) (Additional file 1), but this was not statistically significant (*P* = 0.18, Wilcoxon rank sum test).

In patients with at least one clonal expansion under selective pressure the association between the number of clones and the minimum proximity of the normal samples to the matched tumour was not statistically significant (*P* = 0.307, Wilcoxon rank sum test). Similarly, there was no evidence of an association between the matched tumour being multifocal and the presence of at least one clonal expansion (*P* = 0.79, Wilcoxon rank sum test).

### Mutational signatures

**Fig. 4:**
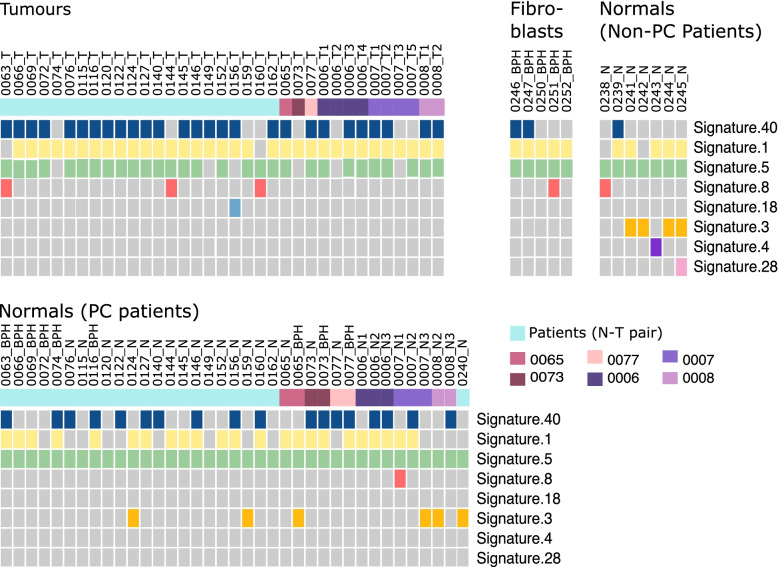
Mutational spectra. Mutational signatures detected in tumour and matched morphologically normal tissue from prostate cancer patients and normal tissue from men without prostate cancer. ﻿The mutational spectra of each sample, as defined by the triplets of nucleotides around each SNV, were deconvoluted into mutational signatures (SigProfiler [41]) using the set of signatures defined by Alexandrov et al. [47]. The colour of the first row indicates patient when there is more than a normal-tumour (N-T) pair analysed. Six patients had more than two samples analysed and one patient had only a morphologically normal sample without a matched tumour

Mutational signatures were inferred for each sample using SigProfiler [41] using the set of signatures defined by Alexandrov et al. [47] (Additional file 6). The cosine similarity between the reference signatures and the reconstructed profiles was high for all samples but higher in tumour compared to normal samples (median of 0.97 for tumour vs 0.88 normal), likely the result of a lower number of SNVs in normal tissues. Mutational signatures 1, 5, 8, 18 and 40 were detected both in tumour and in morphologically normal tissue/BPH samples (Fig. 4). All of these signatures have been previously been identified in prostate cancer samples [47]. Signature 1 was overrepresented in tumour samples (*P* = 4.89 × 10^–03^, Fisher’s exact test). This signature is thought to result from an endogenous mutational process started by the deamination of 5-methylcytosine and has been associated with ageing. Because of this we would expect a similar representation of this signature in both normal and tumour samples. The aetiologies of signatures 5, 8 and signature 40 are unknown [47]. Three signatures (3, 4, and 28) were unique to morphologically normal tissue. Signatures 4 and 28 were present in only one sample, whereas signature 3 is present in 10 samples. Signature 3 has been linked with defective homologous recombination-based repair, Signature 4 has been associated with tobacco smoking and the aetiology of signature 28 is unknown. There were no differences between non-BPH morphologically normal tissue and BPH.

### Gene mutations with functional impact

In morphologically normal, fibroblasts and BPH samples a total of 281 SNVs and indel mutations were observed in coding regions of 165 genes. 110 of the 281 mutations show a potential functional impact according to wANNOVAR [48] and eight of these occurred in known cancer-related genes (*PPARG, BRCA1, GATA1, ACR, WHSC1, FAT1, POLE* and *HOXD11*) as reported in the cancer gene census [49] (Additional file 7). Of these, mutations in *GATA1, WHSC1, ACR,* and *POLE* were observed in at least one sample from a primary prostate fibroblast culture (*WHSC1* and *ACR* occurring in the same sample). Mutations with predicted functional impact were observed in 11 genes that are designated prognostic markers of poor outcome in The Cancer Genome Atlas Research Network (TCGA) RNAseq dataset [50, 51]: *FAT1*, *SOBP, CTHRC1, IQGAP1, FOXJ3, ATP1A3, PHF12, BCAT1, GMPR2, ADAM28*, *DHX32, DSG3, DDX19A, KIAA1217, PPARG, PTK2B, RPL18, DONSON, CHPF2* and *XKRX*. All apart from 4 of the 110 mutations were detected in a single sample: mutations affecting genes *GYPA* and *NACAD* were present in multiple samples from different patients, and mutations in genes *BCAT1* and *FAT2* were present in two samples from the same patient (Additional file 7). Of all the genes identified, only *BRCA2* and *ADAM28* have been previously classified as recurrently mutated drivers in prostate cancer [52, 53]. A previously described dN/dS driver detection method [5] was performed but no significant hits were found, possible due to the limited number of mutations and samples. From the 110 genes with a predicted functional impact, 13 were also observed to be mutated in at least one tumour sample (Additional file 7). However, there was only one instance where a potentially functionally important mutation occurred in both a normal sample and the matched tumour from the same patient (gene *ACOT1* in patient 0122).

We conclude that some of the observed mutations had the potential to generate driver genes but there was an absence of evidence for recurrent mutations in cancer driver genes.

### Comparison with tumours

**Fig. 5:**
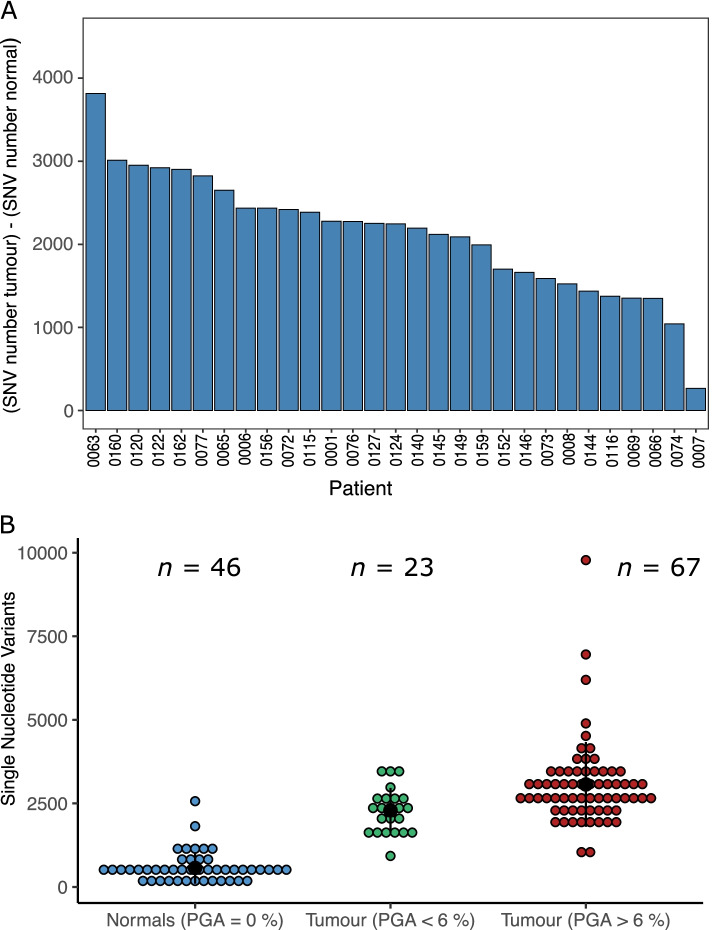
Tumours show a distinct mutation profile to normal tissue. **A** The difference between the number of single nucleotide variants (SNVs) detected in normal tissue compared to tumour tissue. Where multiple samples of either type were present the median number was used. **B** The distribution of the number of SNVs detected in morphologically normal tissue, tumour tissue with low CNAs (percentage genome altered (PGA) < 6%) and tumour tissue with high CNAs (PGA > 6%). Data from these last two categories came from Wedge et al. [52]

When comparing the morphologically normal samples to their respective tumours, both the number of SNVs (median 421 vs 2560.5) and structural rearrangements (median 0 vs 40) was significantly higher in tumours (*P* = 3.73 × 10^–09^, *P* = 2.70 × 10^–06^, respectively, paired Wilcoxon signed rank test; Fig. 5A; Additional file 1). In total 17,370 indels (median of 445) were identified in morphologically normal samples whereas tumour samples harboured 11,087 indels (median of 265). The absence of copy number alterations is a notable characteristic of the normal samples, and the number is significantly less than in cancer tissue (median of 42 for cancer vs 0 for morphologically normal, *P* = 2.68 × 10^–06^, paired Wilcoxon signed rank test).

We analysed a total of 91 of the 112 tumours examined by Wedge et al. [52] (removing the metastatic samples; Additional file 8). A group of 23 samples with less than 6% of the genome affected by copy number alterations were identified as “quiet tumours” (Supplementary Table 3). The numbers of SNVs (median = 2250 vs 2796) and structural rearrangements (median = 32 vs 56) were significantly lower in the quiet tumours than their high CNAs counterparts (*P* = 7.59 × 10^–04^ and *P* = 5.27 × 10^–03^, respectively, Wilcoxon rank sum test). The number of SNVs was significantly higher in “quiet tumours” when compared to samples from morphologically normal tissue (*P* = 1.88 × 10^–10^, Wilcoxon rank sum test, median = 421 vs 2250; Fig. 5B).

## Discussion

Our study demonstrates several critical and recurrent features of the mutations present in non-neoplastic (BPH and non-BPH) tissue taken from cancerous prostates. Primarily, morphologically normal tissue from patients with prostate cancer had a high number of single nucleotide variants (SNVs) and indels and generally a clonal expansion under selective pressure was present. This contrasted with samples from prostates lacking cancer which had a significantly lower number of mutations and a lack of clonal expansions under selective pressure. Our results indicate that the presence of the clonal expansions in non-neoplastic tissue is a feature associated with development of cancer, a finding previously reported in leukemia [14–17].

We also show that there is evidence that clonal expansions from non-neoplastic tissue originates from stromal cells. This is highlighted by the finding that the clonal cell fraction of clonal expansions of morphologically normal tissue was always higher than the proportion of the prostate estimated as epithelial. This is supported by the relationship we observe between non-BPH and BPH normal tissue, with BPH in some cases thought to be associated with hyper-proliferation of stromal tissue [54] (although we found no evidence of an association between stromal content and mutation burden). Firstly, our constructed phylogenies reveal non-BPH morphologically normal and BPH samples within the same prostate can have a shared lineage. Secondly, high mutation rates were observed in five primary cell cultures of stromal cells prepared from BPH specimens; four of the cultures exhibiting evidence of selective clonal expansion; and three samples containing potential driver genes. Thirdly, higher mutation rates were observed in stroma-dominated BPH compared to non-BPH morphologically normal tissue. Finally, the cystoprostatectomy sample 0239 – which exhibited BPH – had the highest number of SNVs observed in non-cancer patients and had evidence of a clonal expansion under selection. The importance of stroma in prostate differentiation was established in mouse studies by Cunha et al. [55]. These studies have been extended into human cells [56–58] and Maitland et al. have studied prostate stromal influences for more than 20 years, exploiting primary clinical material and cultured cells [59, 60]. Foster et al. have reported clonal expansions in cancer-associated fibroblasts (CAFs) [61] and shown that stromal cells from BPH, unlike stromal cells from normal prostatic tissue, have capability of inducing growth of prostatic epithelia in vivo. Taken together, these findings indicate a model for cancer development wherein the presence of clonal expansions of stromal cells supports cancer development and contributes to the field effect. This theory is in agreement with previous reports of an association between BPH and prostate cancer [62–64], although a causal link has not previously been established. If this model is correct, it cannot exclude a role for stroma in non-BPH normal tissue since prostates without BPH also exhibit multifocal disease. Examining the estimated cellular composition of the stroma, derived from single cell sequencing data, in both PC and non-PC donors would further elucidate the differences we observe.

We found only very limited evidence that in normal tissue known genetic drivers were affected by mutations with potential functional impact – only *PPARG, BRCA1, GATA1, HOXD11, WHSC1*, *FAT1* and *POLE* were identified. These genes have been associated with tumour suppression (*BRCA1* and *FAT1)* [65, 66], DNA repair (*POLE)* [67], morphogenesis (*HOXD11*), epigenetic regulation (*WHSC1)* [68], lipid metabolism (*PPARG)* [69] and red blood cell development (*GATA1)* [70]. They have been previously linked with leukemia [71, 72], breast [73–76], bladder [73, 77] colon [78, 79], kidney [80], endometrial [81], head and neck carcinoma [82–84], pancreatic [73] and prostate [85–87] cancers. The low detection of mutations in potential driver genes agrees with a cross tissue study performed by Moore et al. in participants without detected cancer [7] and raises the possible importance of epigenetic alterations in driving clonal expansion. This involvement of epigenetic changes is supported by the reported high hypermethylation levels in genes such as *APC*, *GTSP1* and *RASSF1* in morphologically normal tissue in the prostate [88–91], that have also been shown to be good predictors of cancer development [88–90]. For example, hypermethylation in genes *APC* and *GTSP1* was reported in 95% and 43% respectively in patients with an initial negative biopsy that later developed prostate cancer [88].

Clonal expansions identified in non-neoplastic tissue have a distinct unrelated pattern to those in malignant tissue but are driven by the same processes. Known prostate cancer associated mutational signatures [47, 92] were present in both morphologically normal and tumour tissue, ﻿suggesting that the same mutational processes are driving the clonal expansions in both cases. This is consistent with our own study in a smaller dataset [22] and studies at other cancer sites [93, 94]. Despite this, our constructed phylogenies reveal that clones in morphologically normal samples are of a distinct lineage from those in the tumour and their mutational characteristics are different: normal samples have significantly fewer SNVs, have very few rearrangements and a complete lack of copy number alterations. We observed this difference both with samples from the same prostate in this study and in comparison with “quiet” tumours studied by Wedge et al. [52]*.* Copy number alterations are an important driving feature of prostate cancer and copy number burden has been associated with a poor prognosis [95–97]. Homologous recombination, non-allelic homologous recombination, non-homologous end joining and microhomology-mediated break-induced replication are double stranded break (DSB) repair mechanisms that could result in CNAs, rearrangements and hypermutation [98]. The absence of these three types of genetic alterations in normal samples suggest that this type of DNA damage by DSB and errors in the repairing mechanisms (or both) occur at a lower rate in normal samples and supports the potential increase of replication errors and non-DSB DNA damage produced by endogenous or exogenous environmental factors.

In summary, these results provide further evidence that the whole prostate environment, in particular stromal cells, are involved in the development of prostate cancer and insights into potential genomic evolution mechanisms at very early stages of development. Our findings have implications for treatment (focal therapy) and early detection approaches.

## Supplementary Information

**Additional file 1.** Sample summary.

**Additional file 2: Supplementary Figure 1.** Age vs number of SNVs detected for normal tissue from Prostate cancer patients and non-prostate cancer donors. For the non-cancer donors, the number of SNVs detected is remarkably consistent (range: 104 to 159), apart from one outlier from a cystoprostatectomy with an exceptionally high number of mutations (1202) that is uniquely classified as BPH. There is no significant correlation in the non-cancer donors between age and SNVs (*ρ* = -0.015, *P* = 0.98, Spearman’s correlation; this is retained even when the outlier is included: *ρ* = 0.49, *P* = 0.27). The number of SNVs detected for non-cancer patients is over 100 SNVs lower than all prostate cancer patients except for one prostate cancer outlier, even in the three samples which are of similar age to the prostate cancer cohort. Looking at only samples in the range 50-73 there is a statistically significant difference in the number of SNVs in prostate cancer vs non-prostate cancer donors (*P* = 0.0093; Wilcoxon rank sum test; excluding the normal outlier). **Supplementary Figure 2.** Example density plots of cell cultured fibroblasts and morphologically normal samples from patients where phylogenies could not be reconstructed due to only having one sample per patient or no detected clonal expansions under positive selection. They show the posterior distribution of the fraction of cells bearing a mutation, modelled by a one-dimensional Bayesian Dirichlet process [43]. The median density is indicated by the purple line and 95% confidence intervals by the blue region. The grey histogram shows the observed frequency density of mutations as a function of the fraction of cells bearing the mutation. **Supplementary Figure 3.** Subclonal architecture of patients with morphologically normal and matched tumour (N-T). Phylogenies revealing the relationships between clones for each case. Each coloured line represents a clone/subclone detected in a particular sample. When two or more coloured lines are together, they represent a clone that is found in all the samples represented. The length of the line is proportional to the weighted number of single nucleotide variants present in each clone; the thickness represents the clonal cell fraction associated with that clone (more detail in Additional file 3). Dotted lines are associated with samples that have no evidence of a unique sample specific clone. **Supplementary Figure 4.** The relationship between the clonal cell fraction (CCF) and the type of normal samples. Boxplots showing the distribution of estimated CCF for each clone detected and the type of normal sample (non-BPH normal tissue, normal tissue with BPH and BPH fibroblasts). **Supplementary Table 1.** Summary of patients with multiple normal samples. Patients 0006, 0007 and 0008 have multiple samples from non-BPH normal and tumour tissue and patients 0065, 0073 and 0077 have a sample from non-BPH and BPH normal tissue. **Supplementary Table 2.** The number of mutations in common between normal samples from the same donor. **Supplementary Table 3.** The mutation characteristics of three groups of samples defined by the proportion of genome affected by copy number alterations. Group 1: Tumour samples examined by Wedge et al. [30] with less than 6% of the genome affected by CNAs; Group 2: Tumour samples examined by Wedge et al. [30] with more than 6% of the genome affected by CNAs; and Group 3: normal samples examined in our study where no CNAs were detected. The median number of SNVs and indels are shown for each group.

**Additional file 3.** Subclonal analysis summary in multiple samples: Worksheet 1 summarises the number of clusters and clonal cell fraction for each patient after applying a multidimensional Bayesian Dirichlet process. Worksheet 2 reports the total number of patients included in the subclonal analysis and the location of normal samples in relation to the tumour sample.

**Additional file 4.** Sample summary of samples with clonal expansions under selection pressure.

**Additional file 5.** Proportion of epithelial and stromal contents for each morphologically normal sample.

**Additional file 6.** Mutational signatures in each patient: Results of mutational signature analyses before and after bootstrap.

**Additional file 7.** Mutations in coding regions with functional significance: Functional impact was assessed using wANNOWAR [48].

**Additional file 8.** Sample summary for the comparison of the distribution of the number of SNVs detected in morphologically normal tissue, tumour tissue with low CNAs (percentage genome altered (PGA) <6 %) and tumour tissue with high CNAs (PGA >6 %).

## Data Availability

The datasets generated during the current study are available in the European Genome-Phenome Archive repository, https://ega-archive.org/datasets/EGAD00001000689 and https://ega-archive.org/datasets/EGAD00001004125. The variant calls generated are available from the corresponding author on reasonable request.

## Abbreviations

BPH: Benign prostatic hyperplasia
BWA-SW: Burrows-Wheeler Aligner’s Smith-Waterman Alignment
CAFs: Cancer-associated fibroblasts
CCF: Clonal Cell Fraction
CNAs: Copy number alterations
IQR: Inter-quartile range
Indels: Insertions and deletions
SNVs: Single nucleotide variants
TCGA: The Cancer Genome Atlas Research Network
WGS: Whole Genome DNA Sequencing

## References

[1] Andreoiu M, Cheng L (2010). Multifocal prostate cancer: biologic, prognostic, and therapeutic implications. Hum Pathol.

[2] Nonn L, Ananthanarayanan V, Gann PH (2009). Evidence for field cancerization of the prostate. Prostate.

[3] Zlotta AR, Egawa S, Pushkar D, Govorov A, Kimura T, Kido M (2013). Prevalence of prostate cancer on autopsy: cross-sectional study on unscreened Caucasian and Asian men. J Natl Cancer Inst.

[4] Slaughter DP, Southwick HW, Smejak W (1953). Field cancerization in oral stratified squamous epithelium; clinical implications of multicentric origin. Cancer.

[5] Martincorena I, Roshan A, Gerstung M, Ellis P, Van Loo P, McLaren S (2015). High burden and pervasive positive selection of somatic mutations in normal human skin. Science (80-).

[6] Martincorena I, Fowler JC, Wabik A, Lawson ARJ, Abascal F, Hall MWJ (2018). Somatic mutant clones colonize the human esophagus with age. Science (80-).

[7] Moore L, Cagan A, Coorens THH, Neville MDC, Sanghvi R, Sanders MA (2021). The mutational landscape of human somatic and germline cells. Nature..

[8] Li R, Di L, Li J, Fan W, Liu Y, Guo W, et al. A body map of somatic mutagenesis in morphologically normal human tissues. Nature. Springer US; 2021; Available from: 10.1038/s41586-021-03836-1 10.1038/s41586-021-03836-1 34433965

[9] Tang J, Fewings E, Chang D, Zeng H, Liu S, Jorapur A (2020). The genomic landscapes of individual melanocytes from human skin. Nature.

[10] Lodato MA, Rodin RE, Bohrson CL, Coulter ME, Barton AR, Kwon M (2018). Aging and neurodegeneration are associated with increased mutations in single human neurons. Science (80-).

[11] Brunner SF, Roberts ND, Wylie LA, Moore L, Aitken SJ, Davies SE (2019). Somatic mutations and clonal dynamics in healthy and cirrhotic human liver. Nature.

[12] Yokoyama A, Kakiuchi N, Yoshizato T, Nannya Y, Suzuki H, Takeuchi Y (2019). Age-related remodelling of oesophageal epithelia by mutated cancer drivers. Nature.

[13] Lee-Six H, Olafsson S, Ellis P, Osborne RJ, Sanders MA, Moore L (2019). The landscape of somatic mutation in normal colorectal epithelial cells. Nature.

[14] Jaiswal S, Fontanillas P, Flannick J, Manning A, Grauman PV, Mar BG (2014). Age-Related Clonal Hematopoiesis Associated with Adverse Outcomes. N Engl J Med..

[15] Genovese G, Kähler AK, Handsaker RE, Lindberg J, Rose SA, Bakhoum SF (2014). Clonal Hematopoiesis and Blood-Cancer Risk Inferred from Blood DNA Sequence. N Engl J Med.

[16] Xie M, Lu C, Wang J, McLellan MD, Johnson KJ, Wendl MC (2014). Age-related mutations associated with clonal hematopoietic expansion and malignancies. Nat Med.

[17] Zink F, Stacey SN, Norddahl GL, Frigge ML, Magnusson OT, Jonsdottir I (2017). Clonal hematopoiesis, with and without candidate driver mutations, is common in the elderly. Blood.

[18] Yizhak K, Aguet F, Kim J, Hess J, Kubler K, Grimsby J, et al. A comprehensive analysis of RNA sequences reveals macroscopic somatic clonal expansion across normal tissues. 2018;416339. Available from: https://www.biorxiv.org/content/early/2018/09/13/416339. 10.1126/science.aaw0726 PMC7350423 31171663

[19] Colom B, Herms A, Dentro S, King C, Sood R, Alcolea M, et al. Precancer: Mutant clones in normal epithelium outcompete and eliminate esophageal micro-tumors. bioRxiv. 2021;2021.06.25.449880. Available from: http://biorxiv.org/content/early/2021/06/25/2021.06.25.449880.abstract 10.1038/s41586-021-03965-7 PMC7612642 34646013

[20] Shoag J, Barbieri CE (2016). Clinical variability and molecular heterogeneity in prostate cancer. Asian J Androl.

[21] Svensson M a, LaFargue CJ, MacDonald TY, Pflueger D, Kitabayashi N, Santa-Cruz AM (2011). Testing mutual exclusivity of ETS rearranged prostate cancer. Lab Invest.

[22] Cooper CS, Eeles R, Wedge DC, Van Loo P, Gundem G, Alexandrov LB (2015). Analysis of the genetic phylogeny of multifocal prostate cancer identifies multiple independent clonal expansions in neoplastic and morphologically normal prostate tissue. Nat Genet.

[23] Parr RL, Dakubo GD, Crandall KA, Maki J, Reguly B, Aguirre A (2006). Somatic mitochondrial DNA mutations in prostate cancer and normal appearing adjacent glands in comparison to age-matched prostate samples without malignant histology. J Mol Diagn.

[24] Grossmann S, Hooks Y, Wilson L, Moore L, O’Neill L, Martincorena I (2021). Development, maturation, and maintenance of human prostate inferred from somatic mutations. Cell Stem Cell.

[25] Chandran UR, Dhir R, Ma C, Michalopoulos G, Becich M, Gilbertson J (2005). Differences in gene expression in prostate cancer, normal appearing prostate tissue adjacent to cancer and prostate tissue from cancer free organ donors. BMC Cancer.

[26] Yu YP, Landsittel D, Jing L, Nelson J, Ren B, Liu L (2004). Gene Expression Alterations in Prostate Cancer Predicting Tumor Aggression and Preceding Development of Malignancy. J Clin Oncol.

[27] Luo JH, Ding Y, Chen R, Michalopoulos G, Nelson J, Tseng G (2013). Genome-wide methylation analysis of prostate tissues reveals global methylation patterns of prostate cancer. Am J Pathol.

[28] Yang B, Bhusari S, Kueck J, Weeratunga P, Wagner J, Leverson G (2013). Methylation profiling defines an extensive field defect in histologically normal prostate tissues associated with prostate cancer. Neoplasia (United States).

[29] Warren AY, Whitaker HC, Haynes B, Sangan T, Mcduffus L, Kay JD (2013). Method for SamplingTissue for ResearchWhich Preserves Pathological Data in Radical Prostatectomy. Prostate.

[30] Cussenot O, Berthon P, Cochand-Priollet B, Maitland NJ, Le Duc A (1994). Immunocytochemical comparison of cultured normal epithelial prostatic cells with prostatic tissue sections. Exp Cell Res.

[31] Pellacani D, Droop AP, Frame FM, Simms MS, Mann VM, Collins AT (2018). Phenotype-independent DNA methylation changes in prostate cancer. Br J Cancer.

[32] Li H, Durbin R (2010). Fast and accurate long-read alignment with Burrows-Wheeler transform. Bioinformatics.

[33] Li H, Durbin R (2009). Fast and accurate short read alignment with Burrows-Wheeler transform. Bioinformatics.

[34] Jones D, Raine KM, Davies H, Tarpey PS, Butler AP, Teague JW (2016). cgpCaVEManWrapper: Simple Execution of CaVEMan in Order to Detect Somatic Single Nucleotide Variants in NGS Data. Curr Protoc Bioinformatics.

[35] Nik-Zainal S, Alexandrov LB, Wedge DC, Van Loo P, Greenman CD, Raine K (2012). Mutational Processes Molding the Genomes of 21 Breast Cancers. Cell.

[36] Alioto TS, Buchhalter I, Derdak S, Hutter B, Eldridge MD, Hovig E (2015). A comprehensive assessment of somatic mutation detection in cancer using whole-genome sequencing. Nat Commun.

[37] Raine KM, Hinton J, Butler AP, Teague JW, Davies H, Tarpey P (2015). cgpPindel: Identifying Somatically Acquired Insertion and Deletion Events from Paired End Sequencing. Curr Protoc Bioinformatics.

[38] Zerbino DR, Birney E (2008). Velvet: algorithms for de novo short read assembly using de Bruijn graphs. Genome Res.

[39] Nik-Zainal S, Van Loo P, Wedge DC, Alexandrov LB, Greenman CD, Lau KW (2012). The life history of 21 breast cancers. Cell.

[40] Alexandrov LB, Kim J, Haradhvala NJ, Huang MN, Tian Ng AW, Wu Y, et al. The repertoire of mutational signatures in human cancer. Nature. Nature Publishing Group; 2020;578:94–101. [cited 2022 Sep 8]. Available from: https://www.nature.com/articles/s41586-020-1943-3. 10.1038/s41586-020-1943-3 PMC7054213 32025018

[41] Bergstrom EN, Huang MN, Mahto U, Barnes M, Stratton MR, Rozen SG (2019). SigProfilerMatrixGenerator: a tool for visualizing and exploring patterns of small mutational events. BMC Genomics.

[42] Rosenthal R, McGranahan N, Herrero J, Taylor BS, Swanton C (2016). deconstructSigs: delineating mutational processes in single tumors distinguishes DNA repair deficiencies and patterns of carcinoma evolution. Genome Biol.

[43] Bolli N, Avet-Loiseau H, Wedge DC, Van Loo P, Alexandrov LB, Martincorena I (2014). Heterogeneity of genomic evolution and mutational profiles in multiple myeloma. Nat Commun.

[44] Gundem G, Van Loo P, Kremeyer B, Alexandrov LB, Tubio JMC, Papaemmanuil E, et al. The evolutionary history of lethal metastatic prostate cancer. Nature. 2015;520:353–7. Available from: http://www.nature.com/doifinder/10.1038/nature14347. 10.1038/nature14347 PMC4413032 25830880

[45] Williams MJ, Werner B, Barnes CP, Graham TA, Sottoriva A (2016). Identification of neutral tumor evolution across cancer types. Nat Genet.

[46] Jiao W, Vembu S, Deshwar AG, Stein L, Morris Q (2014). Inferring clonal evolution of tumors from single nucleotide somatic mutations. BMC Bioinformatics.

[47] Alexandrov LB, Kim J, Haradhvala NJ, Huang MN, Ng AW, Boot A, et al. The Repertoire of Mutational Signatures in Human Cancer. bioRxiv. Cold Spring Harbor Laboratory; 2018 [cited 2019 Sep 30];322859. Available from: 10.1101/322859v1

[48] Chang X, Wang K (2012). wANNOVAR: annotating genetic variants for personal genomes via the web. J Med Genet.

[49] Sondka Z, Bamford S, Cole CG, Ward SA, Dunham I, Forbes SA (2018). The COSMIC Cancer Gene Census: describing genetic dysfunction across all human cancers. Nat Rev Cancer.

[50] Network CGAR, Weinstein JN, Collisson EA, Mills GB, Shaw KRM, Ozenberger BA (2013). The Cancer Genome Atlas Pan-Cancer analysis project. Nat Genet.

[51] Uhlén M, Fagerberg L, Hallström BM, Lindskog C, Oksvold P, Mardinoglu A, et al. Tissue-based map of the human proteome. Science (80- ). American Association for the Advancement of Science; 2015 [cited 2022 Feb 1];347. Available from: 10.1126/science.1260419 10.1126/science.1260419 25613900

[52] Wedge DC, Gundem G, Mitchell T, Woodcock DJ, Martincorena I, Ghori M (2018). Sequencing of prostate cancers identifies new cancer genes, routes of progression and drug targets. Nat Genet.

[53] Armenia J, Wankowicz SAMM, Liu D, Gao J, Kundra R, Reznik E (2018). The long tail of oncogenic drivers in prostate cancer. Nat Genet.

[54] Barclay WW, Woodruff RD, Hall MC, Cramer SD (2005). A system for studying epithelial-stromal interactions reveals distinct inductive abilities of stromal cells from benign prostatic hyperplasia and prostate cancer. Endocrinology.

[55] Cunha GR, Lung B (1979). The importance of stroma in morphogenesis and functional activity of urogenital epithelium. In Vitro.

[56] Chung LW, Davies R (1996). Prostate epithelial differentiation is dictated by its surrounding stroma. Mol Biol Rep.

[57] Cunha GR, Hayward SW, Wang YZ, Ricke WA (2003). Role of the stromal microenvironment in carcinogenesis of the prostate. Int J Cancer.

[58] Zhao H, Peehl DM (2009). Tumor-promoting phenotype of CD90 hi prostate cancer-associated fibroblasts. Prostate.

[59] Lang SH, Stark M, Collins A, Paul AB, Stower MJ, Maitland NJ (2001). Experimental prostate epithelial morphogenesis in response to stroma and three-dimensional matrigel culture. Cell Growth Differ.

[60] Hall JA, Maitland NJ, Stower M, Lang SH (2002). Primary prostate stromal cells modulate the morphology and migration of primary prostate epithelial cells in type 1 collagen gels. Cancer Res.

[61] Foster DS, Ransom RC, Jones RE, Salhotra A, Hu MS, Longaker MT (2018). Abstract 10: Characterizing the Clonal Nature of Cancer Associated Fibroblasts. Plast Reconstr Surg Glob Open.

[62] Ørsted DD, Bojesen SE (2013). The link between benign prostatic hyperplasia and prostate cancer. Nat Rev Urol.

[63] Miah S, Catto J (2014). BPH and prostate cancer risk. Indian J Urol.

[64] Chokkalingam AP, Gridley G, Mclaughlin JK, Adami H (2003). Prostate Carcinoma Risk Subsequent to Diagnosis of Benign Prostatic Hyperplasia A Population-Based Cohort Study in Sweden.

[65] Silver DP, Livingston DM (2012). Mechanisms of BRCA1 tumor suppression. Cancer Discov.

[66] Katoh M (2012). Function and cancer genomics of FAT family genes (review). Int J Oncol.

[67] Ogi T, Limsirichaikul S, Overmeer R, Volker M, Takenaka K, Cloney R (2010). Three DNA Polymerases, Recruited by Different Mechanisms, Carry Out NER Repair Synthesis in Human Cells. Mol Cell.

[68] Campos-Sanchez E, Deleyto-Seldas N, Dominguez V, Carrillo-de-Santa-Pau E, Ura K, Rocha PP (2017). Wolf-Hirschhorn Syndrome Candidate 1 Is Necessary for Correct Hematopoietic and B Cell Development. Cell Rep.

[69] Lefterova MI, Zhang Y, Steger DJ, Schupp M, Schug J, Cristancho A (2008). PPARgamma and C/EBP factors orchestrate adipocyte biology via adjacent binding on a genome-wide scale. Genes Dev.

[70] Ferreira R, Ohneda K, Yamamoto M, Philipsen S (2005). GATA1 function, a paradigm for transcription factors in hematopoiesis. Mol Cell Biol.

[71] Shimizu R, Engel JD, Yamamoto M (2008). GATA1-related leukaemias. Nat Rev Cancer.

[72] Swaroop A, Oyer JA, Will CM, Huang X, Yu W, Troche C (2019). An activating mutation of the NSD2 histone methyltransferase drives oncogenic reprogramming in acute lymphocytic leukemia. Oncogene.

[73] Consortium APG (2017). AACR Project GENIE: Powering Precision Medicine through an International Consortium. Cancer Discov.

[74] Rosen EM, Fan S, Pestell RG, Goldberg ID (2003). BRCA1 gene in breast cancer. J Cell Physiol.

[75] Cao XH, Lv JX, Wei XY, Ltuamba EDGL, Hu HL, Zhang YN (2017). FAT1 expression in different breast lesions and its down-regulation in breast cancer development. Int J Clin Exp Pathol.

[76] Yu S, Jiang X, Li J, Li C, Guo M, Ye F (2019). Comprehensive analysis of the GATA transcription factor gene family in breast carcinoma using gene microarrays, online databases and integrated bioinformatics. Sci Rep.

[77] Cazier J-B, Rao SR, McLean CM, Walker AK, Walker AL, Wright BJ (2014). Whole-genome sequencing of bladder cancers reveals somatic CDKN1A mutations and clinicopathological associations with mutation burden. Nat Commun.

[78] Guerra J, Pinto C, Pinto D, Pinheiro M, Silva R, Peixoto A (2017). POLE somatic mutations in advanced colorectal cancer. Cancer Med.

[79] Yu J, Liu M, Liu H, Zhou L (2019). GATA1 promotes colorectal cancer cell proliferation, migration and invasion via activating AKT signaling pathway. Mol Cell Biochem.

[80] Peters I, Dubrowinskaja N, Tezval H, Kramer MW (2015). Decreased mRNA expression of GATA1 and GATA2 is associated with tumor aggressiveness and poor outcome in clear cell renal cell carcinoma.

[81] Imboden S, Nastic D, Ghaderi M, Rydberg F, Rau TT, Mueller MD (2019). Phenotype of POLE-mutated endometrial cancer. PLoS ONE.

[82] Saloura V, Cho H-S, Kiyotani K, Alachkar H, Zuo Z, Nakakido M (2015). WHSC1 Promotes Oncogenesis through Regulation of NIMA-Related Kinase-7 in Squamous Cell Carcinoma of the Head and Neck. Mol Cancer Res.

[83] Lin SC, Lin LH, Yu SY, Kao SY, Chang KW, Cheng HW (2018). FAT1 somatic mutations in head and neck carcinoma are associated with tumor progression and survival. Carcinogenesis.

[84] de Barros e Lima Bueno R, Ramão A, Pinheiro DG, Alves CP, Kannen V, Jungbluth AA (2016). HOX genes: potential candidates for the progression of laryngeal squamous cell carcinoma. Tumor Biol.

[85] Castro E, Eeles R (2012). The role of BRCA1 and BRCA2 in prostate cancer. Asian J Androl.

[86] Li N, Xue W, Yuan H, Dong B, Ding Y, Liu Y (2017). AKT-mediated stabilization of histone methyltransferase WHSC1 promotes prostate cancer metastasis. J Clin Invest.

[87] Elix C, Pal SK, Jones JO (2018). The role of peroxisome proliferator-activated receptor gamma in prostate cancer. Asian J Androl.

[88] Trock BJ, Brotzman MJ, Mangold LA, Bigley JW, Epstein JI, McLeod D (2012). Evaluation of GSTP1 and APC methylation as indicators for repeat biopsy in a high-risk cohort of men with negative initial prostate biopsies. BJU Int.

[89] Partin AW, Van Neste L, Klein EA, Marks LS, Gee JR, Troyer DA (2014). Clinical validation of an epigenetic assay to predict negative histopathological results in repeat prostate biopsies. J Urol.

[90] Stewart GD, Leander VN, Philippe D, Paul D, Agnès D, Alan MS (2013). Clinical Utility of an Epigenetic Assay to Detect Occult Prostate Cancer in Histopathologically Negative Biopsies: Results of the MATLOC Study. J Urol.

[91] Henrique R, Jerónimo C, Teixeira MR, Hoque MO, Carvalho AL, Pais I (2006). Epigenetic Heterogeneity of High-Grade Prostatic Intraepithelial Neoplasia: Clues for Clonal Progression in Prostate Carcinogenesis. Mol Cancer Res.

[92] Alexandrov LB, Kim J, Haradhvala NJ, Huang MN, Tian Ng AW, Wu Y (2020). The repertoire of mutational signatures in human cancer. Nature.

[93] Blokzijl F, de Ligt J, Jager M, Sasselli V, Roerink S, Sasaki N (2016). Tissue-specific mutation accumulation in human adult stem cells during life. Nature.

[94] Ju YS, Martincorena I, Gerstung M, Petljak M, Alexandrov LB, Rahbari R (2017). dynamics in the early human embryo. Nat Publ Gr.

[95] Camacho N, Van Loo P, Edwards S, Kay JD, Matthews L, Haase K (2017). Appraising the relevance of DNA copy number loss and gain in prostate cancer using whole genome DNA sequence data. PLoS Genet.

[96] Taylor BS, Schultz N, Hieronymus H, Gopalan A, Xiao Y, Carver BS (2010). Integrative Genomic Profiling of Human Prostate Cancer. Cancer Cell.

[97] Hieronymus H, Schultz N, Gopalan A, Carver BS, Chang MT, Xiao Y (2014). Copy number alteration burden predicts prostate cancer relapse. Proc Natl Acad Sci U S A.

[98] Ponder RG, Fonville NC, Rosenberg SM (2005). A switch from high-fidelity to error-prone DNA double-strand break repair underlies stress-induced mutation. Mol Cell.

