# Supplementary material for: The architecture of clonal expansions in morphologically normal tissue from cancerous and non-cancerous prostates
Source: Mol Cancer. 2022 Sep 22;21:183. doi: 10.1186/s12943-022-01644-3 (PMC9494848; doi:10.1186/s12943-022-01644-3)
Supplement: Supplementary file 2 — Additional file 2: Supplementary Figure 1. Age vs number of SNVs detected for normal tissue from Prostate cancer patients and non-prostate cancer donors. For the non-cancer donors, the number of SNVs detected is remarkably consistent (range: 104 to 159), apart from one outlier from a cystoprostatectomy with an exceptionally high number of mutations (1202) that is uniquely classified as BPH. There is no significant correlation in the non-cancer donors between age and SNVs (ρ = -0.015, P = 0.98, Spearman’s correlation; this is retained even when the outlier is included: ρ = 0.49, P = 0.27). The number of SNVs detected for non-cancer patients is over 100 SNVs lower than all prostate cancer patients except for one prostate cancer outlier, even in the three samples which are of similar age to the prostate cancer cohort. Looking at only samples in the range 50-73 there is a statistically significant difference in the number of SNVs in prostate cancer vs non-prostate cancer donors (P = 0.0093; Wilcoxon rank sum test; excluding the normal outlier). Supplementary Figure 2. Example density plots of cell cultured fibroblasts and morphologically normal samples from patients where phylogenies could not be reconstructed due to only having one sample per patient or no detected clonal expansions under positive selection. They show the posterior distribution of the fraction of cells bearing a mutation, modelled by a one-dimensional Bayesian Dirichlet process [43]. The median density is indicated by the purple line and 95% confidence intervals by the blue region. The grey histogram shows the observed frequency density of mutations as a function of the fraction of cells bearing the mutation. Supplementary Figure 3. Subclonal architecture of patients with morphologically normal and matched tumour (N-T). Phylogenies revealing the relationships between clones for each case. Each coloured line represents a clone/subclone detected in a particular sample. When two or more coloured lines are [file 12943_2022_1644_MOESM2_ESM.docx]

**Supplementary Table 1. Summary of patients with multiple normal samples.** Patients 0006, 0007 and 0008 have multiple samples from non-BPH normal and tumour tissue and patients 0065, 0073 and 0077 have a sample from non-BPH and BPH normal tissue.

| **Patient** | **Sample type** | **Number of samples** |
| --- | --- | --- |
| 0006 | Tumour | 4 |
|  | Non-BPH normal | 3 |
| 0007 | Tumour | 5 |
|  | Non-BPH normal | 3 |
| 0008 | Tumour | 3 |
|  | Non-BPH normal | 3 |
| 0065 | Non-BPH normal | 1 |
|  | BPH normal | 1 |
| 0073 | Non-BPH normal | 1 |
|  | BPH normal | 1 |
| 0077 | Non-BPH normal | 1 |
|  | BPH normal | 1 |

**Supplementary Table 2. The number of mutations in common between normal samples from the same donor.**

| **Donor** | **Samples** | **SNVs** | **Indels** | **SVs** |
| --- | --- | --- | --- | --- |
| 0006 | N1;N2 | 25 | 4 | 0 |
| 0006 | N1;N3 | 1 | 1 | 0 |
| 0006 | N2;N3 | 125 | 208 | 0 |
| 0006 | N1;N2;N3 | 0 | 2 | 0 |
| 0007 | N1;N2 | 622 | 4 | 0 |
| 0007 | N1;N3 | 2 | 2 | 0 |
| 0007 | N2;N3 | 221 | 284 | 0 |
| 0007 | N1;N2;N3 | 1 | 2 | 0 |
| 0008 | N1;N2 | 0 | 0 | 0 |
| 0008 | N1;N3 | 0 | 0 | 0 |
| 0008 | N2;N3 | 199 | 265 | 0 |
| 0065 | N;BPH | 129 | 188 | 0 |
| 0073 | N;BPH | 94 | 282 | 0 |
| 0077 | N;BPH | 201 | 0 | 0 |

**Supplementary Table 3.** **The mutation characteristics of three groups of samples defined by the proportion of genome affected by copy number alterations**. Group 1: Tumour samples examined by Wedge et al.^30^ with less than 6% of the genome affected by CNAs; Group 2: Tumour samples examined by Wedge et al.^30^ with more than 6% of the genome affected by CNAs; and Group 3: normal samples examined in our study where no CNAs were detected. The median number of SNVs and indels are shown for each group.

| **% of genome affected by CNAs** | **N^0^ samples** | **Median SNVs** | **Median indels** | **Samples** |
| --- | --- | --- | --- | --- |
| > 6 | 67 | 2796 | 2576 | Tumour samples from Wedge *et al*.^29^ |
| < 6 | 23 | 2250 | 2125 | Tumour samples from Wedge *et al*.^29^ |
| 0 | 44 | 421 | 445 | Normal samples from WGS |

**Supplementary Figure 1:** Age vs number of SNVs detected for normal tissue from Prostate cancer patients and non-prostate cancer donors. For the non-cancer donors, the number of SNVs detected is remarkably consistent (range: 104 to 159), apart from one outlier from a cystoprostatectomy with an exceptionally high number of mutations (1202) that is uniquely classified as BPH. There is no significant correlation in the non-cancer donors between age and SNVs (ρ = -0.015, P = 0.98, Spearman’s correlation; this is retained even when the outlier is included: ρ = 0.49, P = 0.27). The number of SNVs detected for non-cancer patients is over 100 SNVs lower than all prostate cancer patients except for one prostate cancer outlier, even in the three samples which are of similar age to the prostate cancer cohort. Looking at only samples in the range 50-73 there is a statistically significant difference in the number of SNVs in prostate cancer vs non-prostate cancer donors (P = 0.0093; Wilcoxon rank sum test; excluding the normal outlier).


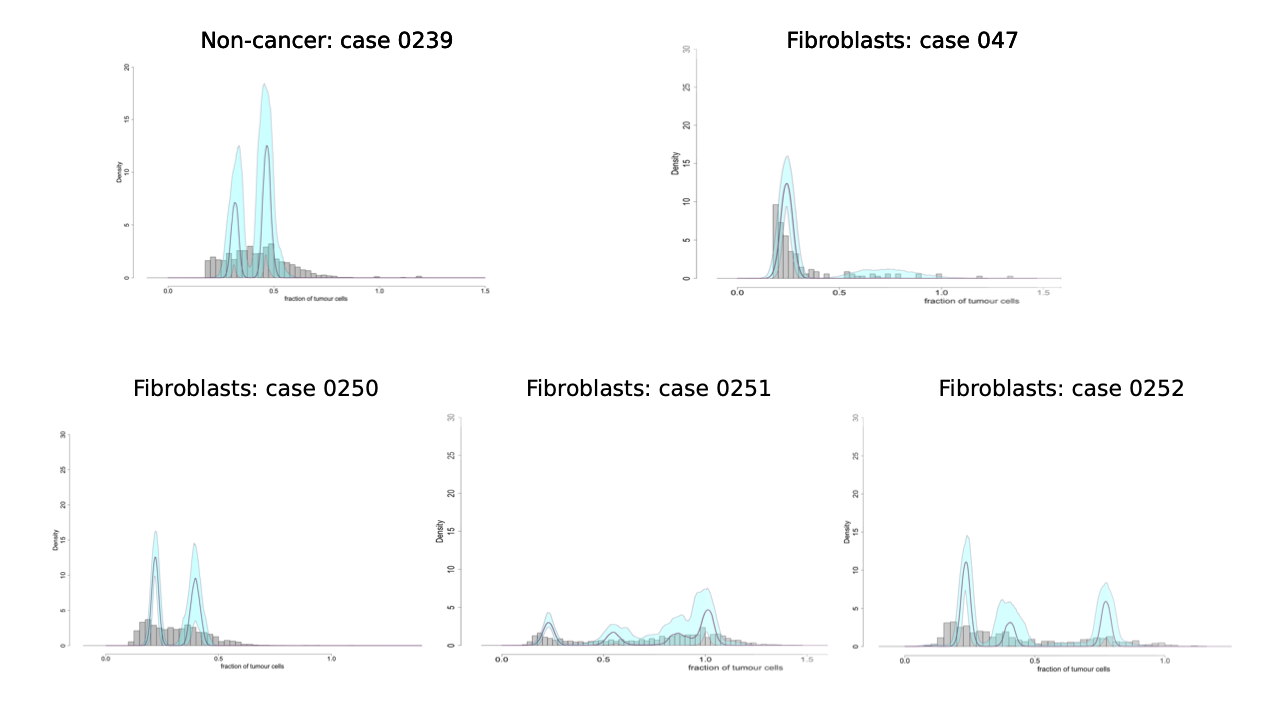


**Supplementary Figure 2*:*** Example density plots of cell cultured fibroblasts and morphologically normal samples from patients where phylogenies could not be reconstructed. They show the posterior distribution of the fraction of cells bearing a mutation, modelled by a one-dimensional Bayesian Dirichlet process^21^. The median density is indicated by the purple line and 95% confidence intervals by the blue region. The grey histogram shows the observed frequency density of mutations as a function of the fraction of cells bearing the mutation.


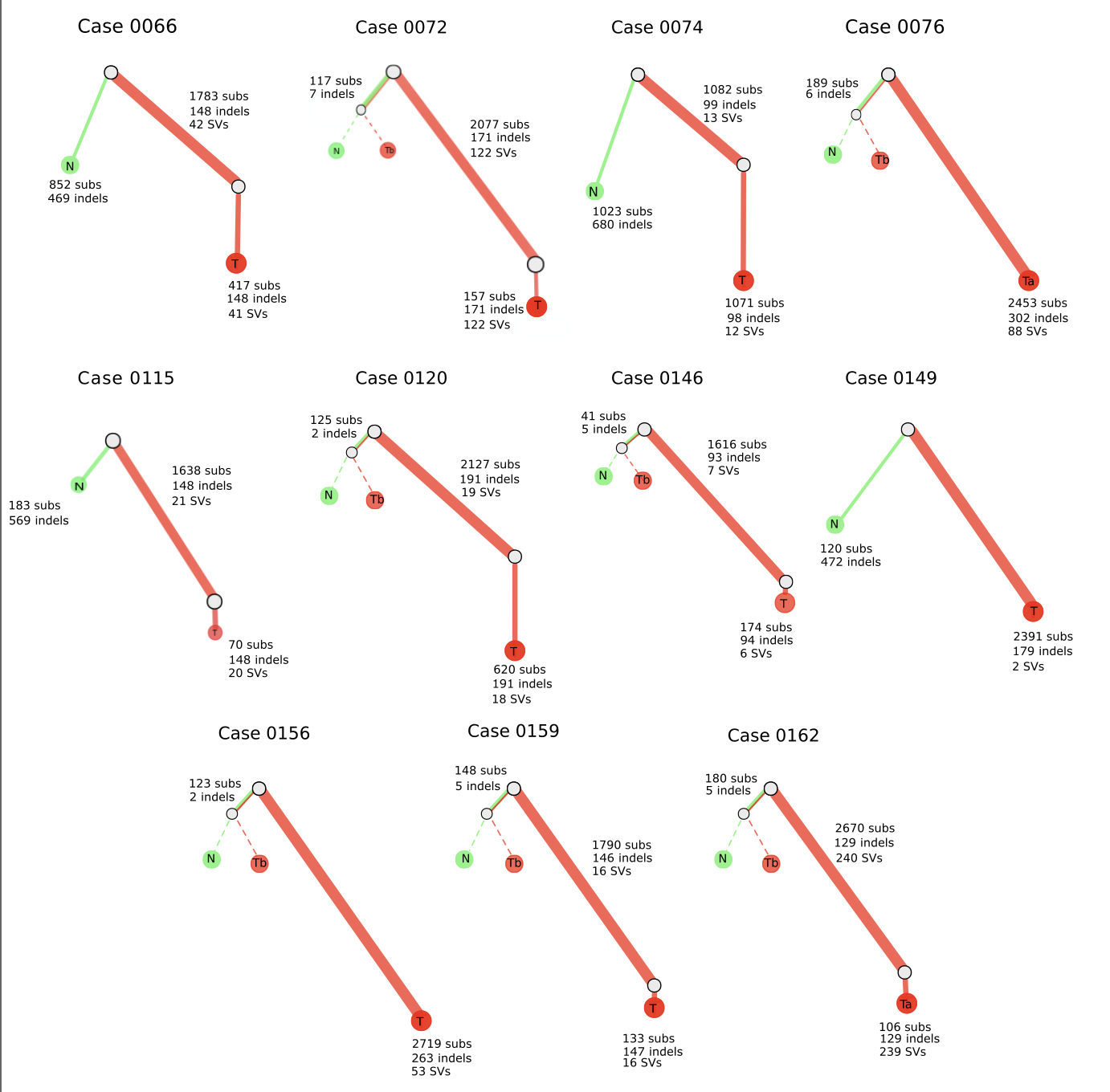


**Supplementary Figure 3. Subclonal architecture of patients with morphologically normal and matched tumour (N-T).** Phylogenies revealing the relationships between clones for each case. Each coloured line represents a clone/subclone detected in a particular sample. When two or more coloured lines are together, they represent a clone that is found in all the samples represented. The length of the line is proportional to the weighted number of substitutions present in each clone; the thickness represents the cell fraction associated with that clone (more detail in Supplementary Table 3). Dotted lines are associated with samples that have no evidence of a unique sample specific clone.

**Supplementary Figure 4.** **The relationship between the cellular cell fraction (CCF) and the type of normal samples.** Boxplots showing the distribution of estimated CCF for each clone detected and the type of normal sample (non-BPH normal tissue, normal tissue with BPH and BPH fibroblasts).
